# The state of boredom: Frustrating or depressing?

**DOI:** 10.1007/s11031-018-9710-6

**Published:** 2018-07-06

**Authors:** Edwin A. J. van Hooft, Madelon L. M. van Hooff

**Affiliations:** 1 Work and Organizational Psychology, University of Amsterdam, P.O. Box 15919, 1001 NK Amsterdam, The Netherlands; 2 Behavioural Science Institute, Radboud University Nijmegen, Nijmegen, The Netherlands

**Keywords:** Emotions, Boredom, Frustration, Depressed affect, Autonomy

## Abstract

Boredom is a prevalent emotion with potential negative consequences. Previous research has associated boredom with outcomes indicating both high and low levels of arousal and activation. In the present study we propose that the situational context is an important factor that may determine whether boredom relates to high versus low arousal/activation reactions. In a correlational (*N* = 443) and an experimental study (*N* = 120) we focused on the situational factor (perceived) task autonomy, and examined whether it explains when boredom is associated with high versus low arousal affective reactions (i.e., frustration versus depressed affect). Results of both studies indicate that when task autonomy is low, state boredom relates to more frustration than when task autonomy is high. In contrast, some support (i.e., Study 1 only) was found suggesting that when task autonomy is high, state boredom relates to more depressed affect than when task autonomy is low. These findings imply that careful attention is needed for tasks that are relatively boring. In order to reduce frustration caused by such tasks, substantial autonomy should be provided, while monitoring that this does not result in increased depressed affect.

## Introduction

Boredom is a prevalent experience, not only among students at school (e.g., Vogel-Walcutt et al. 2012) but also among adults in their daily lives and among employees at work. For example, Harris (2000) found that among students 90% sometimes experience boredom, with a median of one time per day. Among working adults prevalence estimates range from a quarter up to 87% reporting feelings of boredom at work at least sometimes (Fisher 1993; Mann 2007; Van der Heijden et al. 2012; Watt and Hargis 2010). While individual differences exist in the tendency to experience boredom (i.e., boredom proneness; Farmer and Sundberg 1986; Vodanovich 2003; Vodanovich and Kass 1990), boredom can also be viewed as a temporary state, which is often linked to a lack of external stimulation or challenge. Contextual factors such as monotony, repetitiveness, lack of novelty, low task identity, having little to do, and too simple tasks are important causes of boredom (e.g., Fisher in press; Loukidou et al. 2009; Smith 1981; Van Hooff and Van Hooft 2017).

Although boredom may sometimes instigate positive behaviors such as challenge-seeking, reflection, creativity, and prosocial behavior (Carroll et al. 2010; Csikszentmihalyi 1990; Harris 2000; Van Tilburg and Igou 2017b), it more commonly is associated with negative outcomes for individuals, organizations, and society. Examples of such negative outcomes include attention problems (Pekrun et al. 2010; Van Tilburg and Igou 2017a), reduced motivation and effort (Pekrun et al. 2002, 2010), poor performance (Pekrun et al. 2002, 2010), counterproductive behavior (Bruursema et al. 2011; Van Hooff and Van Hooft 2014), property damage (Drory 1982), work injuries (Frone 1998), withdrawal from work (Kass et al. 2001; Reijseger et al. 2013; Spector et al. 2006), political extremism (Van Tilburg and Igou 2016), unhealthy eating (Moynihan et al. 2015), depressed feelings (Game 2007; Goldberg et al. 2011; Sommers and Vodanovich 2000; Van Hooff and Van Hooft 2014, 2016; Van Tilburg and Igou 2017a), dissatisfaction (Game 2007; Kass et al. 2001; Lee 1986; Melamed et al. 1995; Reijseger et al. 2013), frustration (Perkins and Hill 1985; Van Tilburg and Igou 2017a), and distress (Melamed et al. 1995; Van Hooff and Van Hooft 2014).

Although it is relatively clear that boredom is associated with negative reactions, it is less clear when and why boredom links to *what* type of reactions. One distinction in the type of reactions relates to the activation level. Whereas some of boredom’s outcomes indicate low arousal (e.g., dissatisfaction, resignation, depressed feelings), others are more indicative of high arousal (e.g., aggression, frustration, stress, counterproductive behavior). Similarly, there is qualitative evidence that experiential aspects associated with boredom include both high arousal feelings such as restlessness and frustration, and low arousal feelings such as depression, fatigue, and sadness (Fahlman et al. 2013; Goetz and Frenzel 2006; Harris 2000; Martin et al. 2006). Previously coined explanations for these seemingly contradicting findings regarding activation level concern different stages in the boredom experience, different types of boredom, or different characteristics of the boredom-inducing task (Eastwood et al. 2012; Goetz et al. 2014). The present study aims to further increase our understanding of the factors that explain when boredom relates to what kind of arousal-related reactions, by looking at task context. More specifically, in a correlational study (Study 1) and an experimental study (Study 2) we seek to unravel under what task conditions boredom is associated with high versus low arousal negative affective reactions by focusing on task autonomy. We will test the hypothesis that (perceived) task autonomy may explain why boredom sometimes relates to high-arousal affective states and sometimes to low-arousal negative affective states.

We focus on frustration and depressed affect as indicators of negative high- and low-arousal states respectively. In the affect literature, emotional states are generally characterized along two orthogonal axes, namely a ‘pleasure-displeasure’ dimension, and a ‘high arousal-low arousal’ dimension (e.g., Russell 1980; Warr 1990). From this perspective, depressed affect is considered an emotional state characterized by displeasure and low arousal, whereas frustration encompasses displeasure and high arousal (Russell 1980). More specifically, according to the Oxford Dictionary, frustration refers to “feeling or expressing distress and annoyance resulting from an inability to change or achieve something”, and depressed affect refers to the feeling of being “in a state of unhappiness or despondency”.

## The state of boredom

Although academic interest in the concept of boredom dates back to the early 1900s (e.g., Barmack 1938; Münsterberg 1913; Wyatt et al. 1937), reviews have labeled boredom a relatively understudied emotion or neglected concept (Smith 1981; Fisher 1993; Loukidou et al. 2009). Pekrun and colleagues (2010) suggested that this may be caused by the inconspicuous nature of boredom as compared to other negative emotions such as anger and anxiousness. Boredom is usually described as a transient unpleasant affective state, associated with a lack of challenge and stimulation by the task or environment (Fisher 1993; Mikulas and Vodanovich 1993; O’Hanlon 1981; Pekrun et al. 2010). This lack of stimulation may be caused by the ongoing activity being uninteresting, underusing one’s capacities, or simply by having too little to do. Boredom is an activity-related emotion, implying that it disappears when the boredom-evoking activity is abandoned (Pekrun et al. 2010; Van Hooff and Van Hooft 2016). As with other emotions (Scherer 2005), boredom can be characterized by subjective feelings, but also by its cognitive components, bodily symptoms, and action tendencies. Cognitively, boredom is typically associated with perceptions of time passing by slowly, difficulty concentrating, and attention problems (Eastwood et al. 2012; Fisher in press; Harris 2000; Pekrun et al. 2010). Bored people usually have a collapsed upper body, lean their head backwards, and display low and inexpansive bodily movements (Wallbott 1998). Further, boredom is commonly associated with a motivation to cognitively or physically change or escape the situation, for example by daydreaming, mind wandering, or falling asleep (Barmack 1938; Fisher in press; Harris 2000), changing the nature of the task, seeking distraction, or engaging in meaningful behavior (Game 2007; Van der Heijden et al. 2012; Van Tilburg and Igou 2011).

Boredom has been conceptualized as originating from low motivation quality, perceiving little task value, and feeling meaningless (Pekrun et al. 2010; Van Hooff and Van Hooft 2017; Van Tilburg and Igou 2012). Boredom is thus related to activities that feel useless or unchallenging, and that do not serve any purpose towards personally meaningful goals. In other words, boredom may be understood as an emotion that signals lack of progress towards goals that people find important in their lives. Control theory (Carver and Scheier 1990; Carver 2004) distinguishes between two different affective systems that are triggered by goal progress during a self-regulatory process. Specifically, lack of progress may result both in feelings of anxiety/fear (i.e., activating) and sadness/depressed feelings (i.e., deactivating), depending on the goal type or one’s regulatory mode. Because task autonomy is an important contextual factor determining people’s self-regulatory processes (e.g., Deci and Ryan 2000), we suggest that the amount of autonomy that people have or perceive for a task may explain whether high- or low-arousal affective reactions are triggered when a task is boring.

## Task autonomy

Autonomy refers to the decision latitude, influence, freedom, discretion, or (potential) control that people have over when and how tasks are done (e.g., Hackman and Oldham 1980; Karasek 1979; Van Veldhoven et al. 2002). High-autonomy task contexts (i.e., autonomy-supportive environments) provide information, opportunities for participation and choice, and understanding of negative emotions, and minimize external controls, whereas low-autonomy task contexts (i.e., controlling environments) are characterized by minimization of participation, choice, and understanding, and demanding language (Deci et al. 1994; Oliver et al. 2008; Ryan and Deci 2006). Controlling environments thwart people’s innate need for autonomy, which reduces motivation quality (Deci and Ryan 2000) and may evoke reactance in an attempt to reestablish a sense of autonomy (Brehm and Brehm 1981). Previous theory and meta-analytic research on the outcomes of autonomy generally indicate positive motivational effects of autonomy. For example, theorizing on motivation in work settings, such as the Job Characteristics Model (Hackman and Oldham 1980), has described autonomy as a core factor affecting job satisfaction and work motivation, which has been supported in subsequent research (Fried and Ferris 1987). Similarly, the Job Demands-Resources model (Demerouti et al. 2001) classifies autonomy as a job resource, which has been demonstrated to contribute to motivational engagement (Crawford et al. 2010). Also in other contexts such as educational and health settings, autonomy has been shown to positively relate to motivation and engagement (Hagger and Chatzisarantis 2009; Vasquez et al. 2016). Regarding affective and mental health outcomes, high autonomy is generally linked to better psychological health and higher satisfaction, whereas low autonomy is linked to frustration and burnout (e.g., Brehm and Brehm 1981; Luchman and Ganzáles-Morales 2013; Ng et al. 2012; Vasquez et al. 2016). Furthermore, a lack of autonomy or the experience of constraint has been identified has one of the causes of boredom (Fenichel 1951; Fisher 1993; Geiwitz 1966; Reijseger et al. 2013; Van Hooff and Van Hooft 2017; Vodanovich and Kass 1990).

While taking into account the main effects of task autonomy on boredom, in the present study we focus on the moderating role of autonomy on the effects of state boredom. Although research on the interaction between autonomy and boredom is lacking, various theories point at potential moderating effects of autonomy in other unpleasant conditions. For example, self-determination theory states that autonomy importantly affects whether the regulation of uninteresting tasks is internalized, with consequences for motivation and well-being (Deci et al. 1994). Seligman’s (1972) learned helplessness theory proposes that situations with uncontrollable straining events result in anxiety but also induce passivity. Theories on work-related stress (e.g., Demerouti et al. 2001; Karasek 1979) typically view autonomy as a resource that may buffer the negative effects of straining task demands.

When focusing on state boredom, we propose that the degree of task autonomy that the environment provides or that individuals perceive, may influence the reactions to boredom. When feeling bored under low-autonomy conditions, people may feel controlled by the environment, which is associated with experiencing a lack of understanding for their feelings (Deci et al. 1994; Oliver et al. 2008). This may cause people to attribute their boredom externally, resulting in externalized affective responses such as frustration. Furthermore, to the extent that boredom indicates a lack of goal progress, low autonomy likely induces feeling constrained by the external environment in achieving one’s goals. Being blocked in achieving meaningful goals is an important cause of frustration (Spector 1978). For example, attending a dull, irrelevant, and uninteresting lecture may evoke feelings of boredom. When the lecture is required (i.e., low autonomy) and students do not perceive it as useful for their goal attainment, and instead perceive that they are wasting time that they could have spent on more meaningful activities that contribute to goal progress, it likely induces agitation and frustration. This line of reasoning aligns with stress theories and findings, such as Fox et al. (2001) who found some evidence that among individuals who perceive low (but not high) autonomy, straining job demands were associated with sabotage behaviors. It also is consistent with self-determination theory, suggesting that when working on uninteresting tasks, autonomy-thwarting conditions lead to introjected regulation which is associated with heightened tension and anxiety (Deci et al. 1994).

When feeling bored under high-autonomy conditions, people perceive freedom to alter the task circumstances. According to self-determination theory, performing uninteresting tasks under autonomy-supporting conditions leads to increased internalization, such that the task is more integrated in one’s sense of self (Deci et al. 1994). However, when people still feel bored by the task, which associates with experiencing lack of meaningfulness and purpose, they more likely attribute their sense of meaninglessness internally, resulting in internalized affective responses. Because of the perceived freedom and choice, which cause internalized regulation, people may blame themselves rather than others for the lack of goal progress that boredom may signal. Self-blame and attributing failures and lack of goal progress internally are associated with depressive symptoms (e.g., Garnefski et al. 2005; Sweeney et al. 1987). For example, unemployed individuals who feel bored and have high autonomy in deciding what to do, likely experience depressed affect because they experience little purpose and progress towards meaningful goals combined with self-blame, causing feelings of dejection and worthlessness.

Based on these rationales, we theorize that autonomy is an important moderator that may explain whether boredom triggers activating/high-arousal or deactivating/low-arousal affective reactions. Specifically, whereas in situations of low autonomy, boredom more likely relates to feelings of frustration, in situations of high autonomy, boredom more likely relates to depressed feelings.

### Hypothesis 1

The relationship between state boredom and frustration is moderated by (perceived) autonomy, such that it is more positive when (perceived) autonomy is low than when (perceived) autonomy is high.

### Hypothesis 2

The relationship between state boredom and depressed affect is moderated by (perceived) autonomy, such that it is more positive when (perceived) autonomy is high than when (perceived) autonomy is low.

We test these hypotheses in two studies. In Study 1 we examine whether in a naturally occurring situation the experience of boredom is differentially associated with frustration and depressed feelings depending on the level of perceived task autonomy. This study employs a correlational design, using questionnaires to measures the constructs of interest. Study 2 was designed to provide a more rigorous test of the association of boredom, autonomy, and their interaction with frustration and depressed affect, by using a 2 × 2 factorial design in which both state boredom and task autonomy were experimentally manipulated.

## Study 1

### Method

Data were collected among psychology students at a Dutch university. In the first 2 months of the program, all first-year psychology students are required to gain experience with a variety of tests, surveys, and experimental tasks as part of the standard curriculum. In a test session of about 2.25 h students complete a battery of psychological tests and experimental tasks. Because students typically experience these test sessions as rather boring, it provides a perfectly suitable setting to study boredom. At the end of the test session, students completed an evaluation questionnaire, which included our measures of perceived autonomy, state boredom, frustration, and depressed affect (in this order). In total, 444 students started with the test session, of which 443 completed the evaluation questionnaires (68.4% female; *M*_age_ = 19.71, *SD* = 1.83). Questionnaire items were completed digitally, and all items had to be answered.

*Perceived task autonomy* was measured with seven items selected and adapted from Van Veldhoven et al.’s (2002) job autonomy scale. This job autonomy scale is one of the scales of the Questionnaire on the Experience and Evaluation of Work (QEEW), which is a psychometrically sound (i.e., high alpha coefficients and support for unidimensionality of the scales) and widely used instrument to measure job characteristics (Evers et al. 2000; Van Veldhoven et al. 2002). Specifically, the job autonomy scale has 11 items with generally high alpha coefficients (e.g., 0.90 in Van Veldhoven et al. 2002). For the purpose of the present study we selected those items that reflected task characteristics that participants may perceive (some) autonomy on in the current study’s task context (e.g., work pace, order of tasks, task interruptions), and reworded these to fit the study context. The specific items were “I could determine myself how to conduct these tasks”, “I had the idea that I could stop with the tasks if I wanted to”, “I could determine the pace in which I completed the tasks”, “I have the opinion that I had some freedom of choice regarding the execution of the tasks”, “I could influence the order in which I performed the tasks”, “I was in control of what I did this evening”, and “I could decide by myself how much time I spent on each task”. Responses were given on a scale from 1 = *totally disagree* to 5 = *totally agree*. Coefficient alpha was 0.69. This relatively low alpha may have been caused by the reduced scale length and the relatively constrained situation of the present study context.

*State boredom* Lee’s (1986) job boredom measure was used to derive our measure for state boredom. The alpha coefficients of Lee’s job boredom measure are generally high (e.g., 0.93–0.95; Lee 1986) and various studies have supported its validity (see Vodanovich 2003). However, the items reflect both task characteristics that may induce boredom, affective and cognitive aspects of boredom, and potential consequences of boredom (Bruursema et al. 2011; Van Hooff and Van Hooft 2014). Because the present study focused on the experience of state boredom, and similar to previous studies (e.g., Van der Heijden et al. 2012; Van Hooff and Van Hooft 2014), we used a truncated version of Lee’s (1986) measure. Specifically, consistent with the definition of boredom as an unpleasant affective state (e.g., Fisher 1993), only those items were included that reflect the core elements of an emotion (i.e., cognitive components, bodily symptoms, action tendencies, and subjective feelings). Items that confounded boredom and its potential causes (e.g., “Is your work monotonous?”) or consequences (e.g., “Do you become irritable on the job?”) were omitted (cf. Van Hooff and Van Hooft 2014). Previous research has shown support for the validity of such a truncated measure in terms of strong correlations with other validated boredom scales (e.g., *r* = .88 with the Dutch Boredom Scale of Reijseger et al. 2013; see; Van Hooff and Van Hooft 2014). Further, given that boredom is an activity-related emotion, items were rephrased to match the specific task context. The four items read: “I was bored during this test session”, “I found the tasks and tests boring”, “During the test session this evening I thought about doing something else”, and “I felt that the time went by slowly during this test session”. Responses were given on a scale from 1 = *totally disagree* to 5 = *totally agree* (α = 0.82).

*Frustration* and *depressed affect* were measured using graphical scales with a format similar to the subjective-units-of-distress measure often used for distress and anxiety (e.g., Ironson et al. 2002; Ponce et al. 2008; Schmidt and Zvolensky 2007). As a direct measure of frustration and depressed affect, participants were asked to indicate with a sliding bar how frustrated they felt at this moment on a 10 cm line with anchors 0 = *totally not frustrated* and 100 = *the most frustrated I have ever been*, and how “down” they felt at this moment with anchors 0 = *totally not* “down” and 100 = *the most* “down” *I have ever been*. Because scores could (and did) vary between 0 and 100 with increments of 1 these scales provide sensitive measures of frustration and depressed affect, allowing for high score variance (see Cook et al. 2001). To check the validity of these measures we also administered two multiple-item adjectives scales after they completed the graphical scales. Similar to other affect measures (e.g., PANAS; Watson et al. 1988; POMS; McNair et al. 1971) we presented a list of adjectives and asked participants to indicate how they felt at the present moment. Each adjective was rated on a 5-point Likert scale (1 = *totally not* to 5 = *very much so*). The adjectives scale for frustration was composed of terms indicative of high-arousal negative affect similar to frustration (i.e., frustrated, annoyed, irritated, and agitated; α = 0.92). The adjectives scale for depressed affect was composed of terms indicative of low-arousal negative affect similar to feeling depressed (i.e., depressed, washed-out, passive, low in energy, and sad; α = 0.85). These terms were based on other affect measures (e.g., McNair et al. 1971; Watson et al. 1988) and circumplex models of affect (e.g., Barrett and Russell 1998; Russell 1980; Warr 1990). Item responses were averaged for the frustration adjectives and for the depressed affect items to create two scale scores. Supporting the validity of our measures, correlations between the graphical measures and the adjectives scales were 0.82 (*p* < .001) for frustration and 0.70 (*p* < .001) for depressed affect.

Confirmatory factor analysis with LISREL 8.80 demonstrated that when specifying a factor structure with the task autonomy, state boredom, frustration adjectives, and depressed affect adjectives items loading on four separate factors, all item loadings were > 0.30 and significant (*p* < .01) and. Furthermore, this four-factor solution provided a significantly better fit, χ^2^(164) = 747.60, *p* < .001, SRMR = .075, than a three-factor solution with the frustration and depressed affect adjective items loading on the same factor, χ^2^(167) = 1490.75, *p* < .001, SRMR = .110, Δχ^2^(3) = 743.15, *p* < .001, or a one-factor solution with all items loading on the same factor, χ^2^(170) = 2425.78, *p* < .001, SRMR = .150, Δχ^2^(6) = 1678.18, *p* < .001.

## Results and discussion

Table 1 presents descriptives and correlations. The two dependent variables frustration and depressed affect were relatively strongly correlated (*r* = .53, *p* < .001). Such correlation is in line with two-dimensional or circumplex models of emotion (e.g., Barrett and Russell 1998), because although frustration and depressed affect differ on the activation-deactivation axis, they both are unpleasant rather than pleasant emotions.

**Table 1:** Study 1 means, standard deviations, and correlations

Variable		*M*	*SD*	1.	2.	3.	4.	5.	6.	7.
1	Sex (0 = *male*; 1 = *female*)	0.68	0.47							
2	Age	19.71	1.83	− 0.27***						
3	Perceived task autonomy (1–5)	2.70	0.67	− 0.03	− 0.01					
4	State boredom (1–5)	3.07	0.92	− 0.09	− 0.12**	− 0.20***				
5	Frustration (graphic scale; 0–100)	20.30	26.13	− 0.00	− 0.03	− 0.23***	0.34***			
6	Frustration (adjectives scale; 1–5)	1.76	0.96	0.01	− 0.04	− 0.20**	0.38**	0.82**		
7	Depressed affect (graphic scale; 0–100)	19.12	25.21	0.05	0.05	− 0.07	0.15***	0.53***	0.46**	
8	Depressed affect (adjectives scale; 1–5)	2.03	0.90	0.13**	− 0.07	− 0.05	0.18**	0.47**	0.40**	0.70**

*N* = 443. The possible range of the variables is indicated between brackets

**p < .01; ***p < .001

**Fig. 1:**
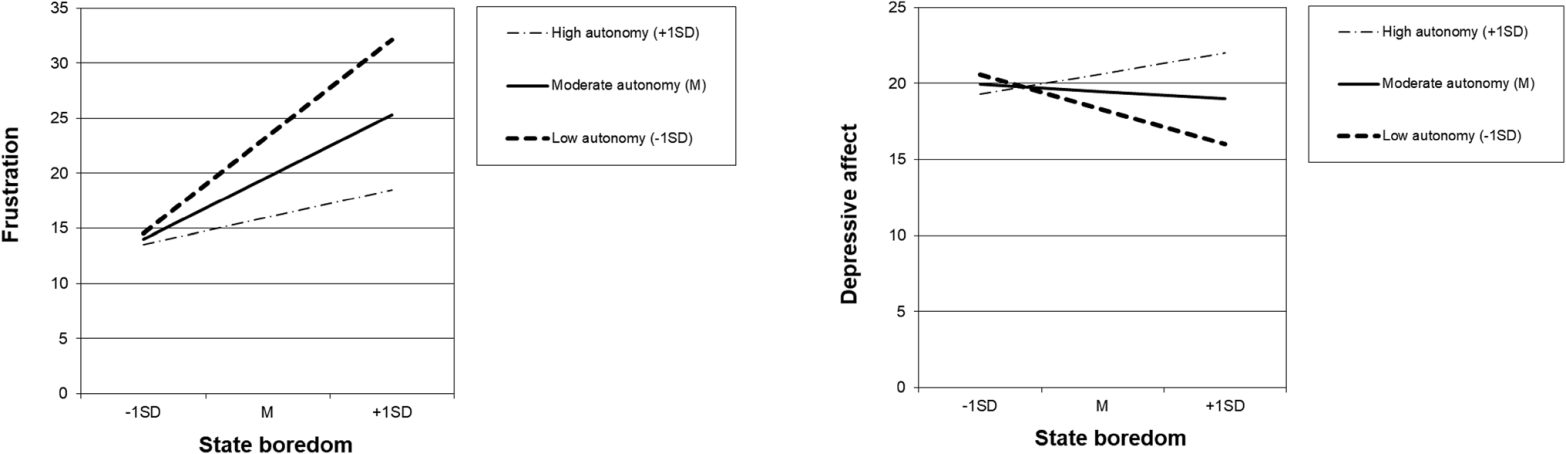
Study 1 simple regression slopes for the association between state boredom and frustration and depressed affect (on scales 0–100) for low (M − 1SD), moderate (M), and high (M + 1SD) levels of perceived task autonomy

Hypothesis 1 proposed that the relationship between boredom and frustration would be moderated by perceived autonomy. We tested our hypothesis with moderated regression analysis, using mean-centered predictor scores to avoid problems of multicollinearity (cf. Aiken and West 1991). Because we were interested in the prediction of frustration independently from depressed affect, we controlled for depressed affect. Specifically, we entered depressed affect, perceived autonomy, and boredom in Step 1 of the regression, and added the interaction between boredom and perceived autonomy in Step 2. As Table 2 displays, autonomy negatively and boredom positively predicted frustration, and the autonomy × boredom interaction was significant. We further analyzed the form of the interaction with simple slopes analyses, using the procedures outlined by Aiken and West (1991). In support of Hypothesis 1, boredom related more positively to frustration at lower autonomy levels (*M* − 1*SD*), *B* = 9.51, *SE* = 1.29, than at higher autonomy levels (*M* + 1*SD*), *B* = 2.70, *SE* = 1.55, *t*(439) = − 3.78, *p* < .001 (see Fig. 1). Whereas the low autonomy slope was significantly positive, *t*(439) = 7.39, *p* < .001, the high autonomy slope was only marginally significant, *t*(439) = 1.74, *p* = .08.

**Table 2:** Study 1 moderated regression analyses of frustration and depressed affect on perceived task autonomy, state boredom, and their interaction

Predictor	Frustration (β)				Depressed affect (β)			
	Graphic scale		Adjectives scale		Graphic scale		Adjectives scale	
	Step 1	Step 2	Step 1	Step 2	Step 1	Step 2	Step 1	Step 2
Perceived task autonomy	− 0.15***	− 0.14***	− 0.12**	− 0.11**	0.05	0.05	0.06	0.06
State boredom	0.24***	0.22***	0.29***	0.27***	− 0.03	− 0.02	0.03	0.04
Depressed affect	0.48***	0.48***	0.40***	0.41***				
Frustration					0.55***	0.56***	0.48***	0.49***
Perceived task autonomy × state boredom		− 0.14***		− 0.11**		0.09*		0.10*
Δ*R*^2^		0.02**		0.01**		0.01*		0.01*
Multiple *R*	0.61***	0.63***	0.56***	0.58***	0.53***	0.54***	0.48***	0.49***
Adjusted *R*^2^	0.37***	0.39***	0.31***	0.33***	0.28***	0.28***	0.22***	0.23***

*N* = 443. The possible range for scores on the graphic scales for frustration and depressed affect was 0–100, and for the scores on the adjectives scales 1–5

*p < .05; **p < .01; ***p < .001

We tested for robustness of our results by repeating the analyses using the adjectives scale for frustration as dependent variable. As displayed in Table 2, similar results were found, with the autonomy × boredom interaction in the same direction and significant at *p* < .01. Simple slopes analyses demonstrated that again in support of Hypothesis 1, boredom related more positively to frustration at lower autonomy levels (*M* − 1*SD*), *B* = 0.38, *SE* = 0.05, than at higher autonomy levels (*M* + 1*SD*), *B* = 0.18, *SE* = 0.06, *t*(439) = − 2.85, *p* < .001, with both the low autonomy slope, *t*(439) = 7.66, *p* < .001, and the high autonomy slope being significantly positive, *t*(439) = 3.04, *p* < .01.

Hypothesis 2 proposed that the relationship between boredom and depressed affect would be moderated by perceived autonomy. This hypothesis was also tested with moderated regression analysis using centered predictor scores, and controlling for frustration to be able to examine the prediction of depressed affect independently from frustration. As shown in Table 2, the main-effect relations of perceived autonomy and boredom with depressed affect were not significant. However, the autonomy × boredom interaction was significant. Analyzing the form of the interaction demonstrates that, in support of Hypothesis 2, boredom related more positively to depressed affect at higher autonomy levels (*M* + 1*SD*), *B* = 1.45, *SE* = 1.62, than at lower autonomy levels (*M* − 1*SD*), *B* = − 2.47, *SE* = 1.42,, *t*(439) = 2.06, *p* < .05 (see Fig. 1). Although both slopes significantly differed from each other as indicated by the significant interaction, the high autonomy slope was not significantly different from zero *t*(439) = 0.90, *p* = .37, and the low autonomy slope was marginally significantly negative, *t*(439) = − 1.74, *p* = .08.

We tested for robustness of our results by repeating the analyses using the adjectives scale for depressed affect as dependent variable. As displayed in Table 2, similar results were found, with the autonomy × boredom interaction in the same direction and significant at *p* < .05. Simple slopes analyses demonstrated that again in support of Hypothesis 2, boredom related more positively to depressed affect at higher autonomy levels (*M* + 1*SD*), *B* = 0.12, *SE* = 0.06, than at lower autonomy levels (*M* − 1*SD*), *B* = − 0.04, *SE* = 0.05, *t*(439) = 2.31, *p* < .05, with the high autonomy slope being significantly positive, *t*(439) = 2.01, *p* < .05, and the low autonomy slope being not significantly different from zero, *t*(439) = − 0.80, *p* = .42.

Altogether these findings provide some first support for our hypotheses, indicating that boredom and perceived task autonomy interact in predicting frustration and depressed affect. Our findings suggest that when people experience boredom it depends on their perceived levels of task autonomy whether they feel more frustrated or more depressed. That is, under conditions of high boredom, people feel more frustration when they perceive *low* rather than high autonomy, and more depressed affect when they perceive *high* rather than low autonomy. Under conditions of low boredom, frustration and depressed affect were not much different depending on the level of perceived autonomy. While this study was conducted in a naturalistic setting with high ecological validity, the study had a correlational design. Therefore, we cannot rule out alternative explanations such as that the relationships are spurious, caused by other unmeasured variables. To rule out this possibility, we conducted a follow-up study using an experimental design.

## Study 2

The second study was designed to provide a more rigorous test of the relationships of boredom, autonomy, and their interaction with frustration and depressed affect. Previous research demonstrated that boredom can be evoked experimentally with boring assignments (e.g., London et al. 1972; Van Tilburg and Igou 2011, 2012). Autonomy has been manipulated before with autonomy-supportive versus controlling task instructions and offering choice or no choice (e.g., Deci et al. 1994; Oliver et al. 2008; Sheldon and Filak 2008). Based on this research, we manipulated boredom and autonomy in a 2 × 2 design to test their hypothesized interactive effects on frustration (Hypothesis 1) and depressed affect (Hypothesis 2).

### Method

#### Participants and design

Participants were recruited at a Dutch university using flyers and online announcements. Based on power analysis, we recruited 120 individuals (73.1% females; *M*_age_ = 21.71, *SD* = 4.77; 44.1% psychology students; one participant failed to complete the demographics), as to reach a power of about 80% to detect medium-sized effects at an α of 0.05 (cf. G*Power; Faul et al. 2007). Participants were randomly assigned to one of four conditions in a 2(high versus low boredom) × 2(high versus low autonomy) between-participants design (*n* = 28 in the high-boredom, high-autonomy condition, *n* = 28 in the high-boredom, low-autonomy condition, *n* = 31 in the low-boredom, high-autonomy condition, and *n* = 33 in the low-boredom, low-autonomy condition). Missing data occurred in case of four participants. Two participants missed one item of a scale (i.e., Time 2 boredom and Time 3 autonomy, respectively). Mean substitution was used for these two missing items. Two other participants failed to complete an entire scale (i.e., Time 3 boredom and Time 3 autonomy, respectively), and one of these participants also failed to complete the demographic items. These participants were excluded from the analyses that involved the respective variables.

#### Procedure

Upon entering the lab, participants were welcomed and received a brochure with general information about the study and signed an informed consent. The information brochure provided information on the purpose of the study (i.e., that the study was about the factors that play a role in people’s emotions), the procedure (i.e., that participants would be asked to complete questionnaires and various tasks), voluntary participation, confidentiality of the data, the rights of the participants, the rewards for participation (i.e., a choice between study credits or €7 cash), and ethical committee contact information. Participants were seated in an individual cubicle and asked to complete a paper-and-pencil baseline questionnaire (Time 1), measuring several personality traits and general trait affectivity. When participants had finished filling in the questionnaire, the experimenter gave them a practice task, which differed depending on the boredom condition that the participants were assigned to. Specifically, participants had to count the number of letters of one versus five (low- vs. high-boredom condition) APA-formatted references. The practice task was used to be able to assess participants’ baseline levels of state boredom (i.e., engaging in an activity is needed to assess the activity-related emotion of boredom), frustration, and depressed affect. These were measured in the second questionnaire (Time 2), along with filler items on interest and other emotions.

Next, participants completed a series of four different tasks, adapted from Van Tilburg and Igou (2011) to manipulate boredom. Specifically, participants had to copy one versus five references, draw lines through three versus nine spirals, draw lines from A to B to C in five versus fifteen boxes, and count the letters of one versus five references. Previous research (Van Tilburg and Igou 2011) has shown that boredom can be induced by increasing the number of trials on each of these tasks (e.g., copying five instead of one reference).

Autonomy was manipulated with the task instructions. Based on previous research that experimentally manipulated autonomy (Deci et al. 1994; Oliver et al. 2008; Sheldon and Filak 2008), the instructions in the high-autonomy condition focused on choice and self-direction and in the low-autonomy condition on no choice and no self-direction. That is, in the high-autonomy condition participants were allowed to determine the task order themselves and the experimenter introduced the tasks in a autonomy-supportive style (cf. Deci et al. 1994; Oliver et al. 2008; Sheldon and Filak 2008). In the low-autonomy condition, the experimenter determined the task order for them and the tasks were introduced in a controlling style (cf. Deci et al. 1994; Oliver et al. 2008; Sheldon and Filak 2008). Specifically, the high-autonomy condition instructions were: “I will now explain the four different tasks. If you have any questions, please feel free to ask. You can choose when you want to start and in which order you want to do the different tasks. Please finish every task before you start a new one. In one task you need to copy [five or one, depending on the boredom condition] references. In another task you need to draw spirals or draw a line between letters in a box. In another task you need to count the letters of [five or one, depending on the boredom condition] references. I will leave the room now and you can start when you feel comfortable.” The low-autonomy condition instructions were: “I explain you what you have to do and I will tell you when to begin. I will give you four different tasks. Do them in the order as they are numbered. [Then the experimenter numbered the order of the tasks in front of participant.] You have to start now.”

After completing the four tasks, participants received a final questionnaire (Time 3) measuring state boredom, frustration, depressed affect, filler items on interest and other emotions, perceived autonomy, and demographics. The experiment took about 45 min. Students were debriefed and received the study credits or €7 for participation.

#### Measures

The dependent variables *frustration* and *depressed affect* were assessed at Time 2 and 3 with the same graphical measures as in Study 1. As manipulation checks we measured participants’ state boredom at Time 2 and 3, and perceived task autonomy at Time 3. *State boredom* was assessed with the same four items from Lee’s (1986) job boredom measure as in Study 1. Items were slightly rephrased to match the specific task context (i.e., “I was bored during the tasks that I just worked on”, “I found the tasks boring”, “During working on the tasks I thought about doing something else”, and “I felt that the time went by slowly during working on the tasks”; α’s were 0.78 and 0.88). *Perceived task autonomy* was measured with three items selected and adapted from the job autonomy scale (Van Veldhoven et al. 2002). Because it served as a manipulation check, we specifically selected items that referred to aspects of autonomy that were targeted in the autonomy manipulation, and thus should display differences between the high and low autonomy conditions if the manipulation had worked. The items were reworded to fit the study context, specifically: “I could determine how I conducted these tasks”, “I could determine the order in which I completed the tasks”, and “I have the opinion that I had some freedom of choice regarding the execution of the tasks” (α = 0.68). Responses on the boredom and autonomy items were given on scales ranging from 1 = *totally disagree* to 5 = *totally agree*.

#### Randomization

To check the successfulness of the randomization, we compared the four conditions on sex, age, personality traits (i.e., boredom proneness with 13 items of the sensation-seeking scale, α = 0.79; self-esteem with ten items of the Rosenberg self-esteem scale, α = 0.90), and general trait affectivity (i.e., positive affectivity, α = 0.79 and negative affectivity, α = 0.85, each with 10 items from the PANAS) measured at Time 1. The percentage of females did not differ significantly across the four conditions, χ^2^(3, *N* = 119) = 3.57, *p* = .31. Furthermore, a two-way MANOVA with age, boredom proneness, self-esteem, positive affectivity, and negative affectivity as dependent variables did not show significant effects of condition boredom, *F*(5, 111) = 0.43, *p* = .82, condition autonomy, *F*(5, 111) = 1.45, *p* = .21, or their interaction, *F*(5, 111) = 0.80, *p* = .55.

## Results

### Manipulation checks

A two-way ANOVA with Time 3 state boredom as dependent variable showed a main effect of condition boredom, *F*(1, 115) = 46.50, *p* < .001, partial η^2^ = 0.29. In support of the manipulation, participants in the high-boredom conditions (*n* = 56) reported a significantly higher level of state boredom at Time 3, *M* = 3.61, *SD* = 1.01, 95% CI [3.37, 3.85], than the participants in the low-boredom conditions (*n* = 63; one participant failed to complete the Time 3 boredom items), *M* = 2.48, *SD* = 0.81, 95% CI [2.25, 2.70]. Whereas the main effect of condition autonomy was not significant, *F*(1, 115) = 1.07, *p* = .30, partial η^2^ = 0.01, the interaction between boredom and autonomy was, *F*(1, 115) = 3.94, *p* < .05, partial η^2^ = 0.03. To further analyze the interaction, subsequent simple effects analyses demonstrate that the boredom manipulation was effective in both autonomy conditions, but a bit more so in the low autonomy condition, *M*_high boredom_ = 3.86 versus *M*_low boredom_ = 2.40, *F*(1, 115) = 39.06, *p* < .001, partial η^2^ = 0.25, than in the high autonomy condition, *M*_high boredom_ = 3.36 versus *M*_low boredom_ = 2.56, *F*(1, 115) = 11.59, *p* < .001, partial η^2^ = 0.09.

A second two-way ANOVA with Time 3 perceived autonomy as dependent variable showed a main effect of condition autonomy, *F*(1, 115) = 97.71, *p* < .001, partial η^2^ = 0.46. In support of the manipulation, participants in the high-autonomy conditions (*n* = 58; one participant failed to complete the Time 3 perceived autonomy items) reported a significantly higher level of perceived autonomy at Time 3, *M* = 3.80, *SD* = 0.69, 95% CI [3.60, 4.01], than the participants in the low-autonomy conditions (*n* = 61), *M* = 2.38, *SD* = 0.88, 95% CI [2.18, 2.58]. Whereas the main effect of condition boredom was not significant, *F*(1, 115) = 0.20, *p* = .65, partial η^2^ = 0.00, the interaction between autonomy and boredom was, *F*(1, 115) = 5.10, *p* < .05, partial η^2^ = 0.04. To further analyze the interaction, subsequent simple effects analyses demonstrate that the autonomy manipulation was effective in both boredom conditions, but a bit more so in the high boredom condition, *M*_high autonomy_ = 3.94 versus *M*_low autonomy_ = 2.19, *F*(1, 115) = 68.55, *p* < .001, partial η^2^ = 0.37, than in the low boredom condition, *M*_high autonomy_ = 3.68 versus *M*_low autonomy_ = 2.58, *F*(1, 115) = 31.46, *p* < .001, partial η^2^ = 0.21.

### Main findings

**Fig. 2:**
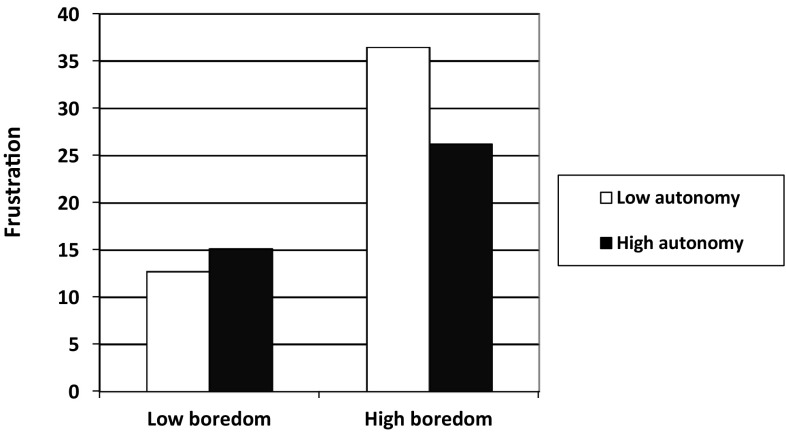
Study 2 estimated marginal means of frustration after the series of four tasks (on a scale 0–100) by boredom (high versus low) and autonomy (high versus low) conditions

Table 3 presents descriptives and correlations and Table 4 presents the means and standard deviations per condition. Hypothesis 1 was tested with a two-way ANCOVA with conditions boredom and autonomy as factors, experiment duration, baseline frustration levels (i.e., at Time 2 after the practice task), and Time 3 depressed affect as covariates, and frustration after the main task series (Time 3) as dependent variable. Results demonstrate a main effect of frustration after the practice task, *F*(1, 115) = 65.93, *p* < .001, partial η^2^ = 0.37, and marginally significant main effects of experiment duration, *F*(1, 115) = 2.92, *p* = .09, partial η^2^ = 0.03, and Time 3 depressed affect, *F*(1, 115) = 3.23, *p* = .07, partial η^2^ = 0.03. Further, the main effect of condition boredom was significant, *F*(1, 115) = 14.41, *p* < .001, partial η^2^ = 0.11, but the main effect of condition autonomy was not, *F*(1, 115) = 2.19, *p* = .14, partial η^2^ = 0.02. Lastly, the boredom × autonomy interaction was significant, *F*(1, 115) = 5.65, *p* < .05, partial η^2^ = 0.05. As displayed in Fig. 2, elevated frustration levels were especially present in the high-boredom low-autonomy condition. In support of Hypothesis 1, subsequent simple effects analyses demonstrate that Time 3 frustration was significantly higher in the high-boredom low-autonomy condition, *M* = 36.47, 95% CI [29.80, 43.14], as compared to the high-boredom high-autonomy condition, *M* = 26.25, 95% CI [19.58, 32.93], *F*(1, 113) = 7.11, *p* < .01. In contrast, the low-boredom low-autonomy condition, *M* = 12.71, 95% CI [6.72, 18.71], and the low-boredom high-autonomy condition, *M* = 15.11, 95% CI [8.82, 21.39], did not differ significantly, *F*(1, 113) = 0.43, *p* = .51.

**Table 3:** Study 2 overall means, standard deviations, and correlations

Variable		*M*	*SD*	1.	2.	3.	4.	5.	6.	7.	8.	9.	10.
1	Sex (0 = *male*; 1 = *female*)	0.73	0.45										
2	Age	21.71	4.77	− 0.09									
3	Experiment duration (*minutes*)	42.60	11.51	0.13	0.00								
4	Condition boredom (0 = *low*; 1 = *high*)	0.47	0.50	0.11	− 0.03	0.83***							
5	Frustration after practice task (0–100)	16.63	17.29	− 0.10	0.19*	0.09	0.07						
6	Depressed affect after practice task (0–100)	14.09	17.38	− 0.13	0.29**	0.03	0.01	0.62***					
7	Condition autonomy (0 = *low*; 1 = *high*)	0.49	0.50	0.14	0.06	− 0.00	0.02	− 0.18*	− 0.20*				
8	State boredom after four tasks (1–5)	3.01	1.07	0.03	− 0.01	0.48***	0.53***	0.30**	0.16	− 0.07			
9	Frustration after four tasks (0–100)	22.03	21.93	− 0.08	0.08	0.22*	0.31**	0.70***	0.44***	− 0.21*	0.48***		
10	Depressed affect after four tasks (0–100)	13.26	16.06	− 0.14	0.17	0.10	0.09	0.59***	0.89***	− 0.16	0.19*	0.53***	
11	Perceived task autonomy (1–5)	3.08	1.06	0.10	− 0.04	0.01	− 0.03	− 0.25**	− 0.25**	0.66***	− 0.21*	− 0.30**	− 0.24**

Due to incidental missing values *N* varies between 118 and 120. The possible range of the variables is indicated between brackets

*p < .05; **p < .01; ***p < .001

**Table 4:** Study 2 means and standard deviations per condition

Variable		*F*	High boredom				Low boredom			
			High autonomy		Low autonomy		High autonomy		Low autonomy	
			*M*	*SD*	*M*	*SD*	*M*	*SD*	*M*	*SD*
1	Sex (0 = *male*; 1 = *female*)	1.18	0.85	0.36	0.71	0.46	0.74	0.44	0.64	0.49
2	Age	0.16	21.74	3.72	21.43	3.35	22.19	4.89	21.48	6.37
3	Experiment duration (*minutes*)	83.07***	52.75_a_	7.17	52.68_a_	7.39	33.39_b_	5.97	34.09_b_	5.79
4	Frustration after practice task (0–100)	2.45	17.54	17.98	18.29	16.15	9.84	11.83	20.85	20.53
5	Depressed affect after practice task (0–100)	1.74	9.96	14.78	18.57	14.84	11.03	17.98	16.67	20.05
6	State boredom after four tasks (1–5)	17.13***	3.36_a_	1.05	3.86_b_	0.93	2.56_c_	0.87	2.40_c_	0.76
7	Frustration after four tasks (0–100)	6.33***	23.18_a_	19.50	35.14_b_	25.42	12.35_c_	17.55	19.03_ac_	19.53
8	Depressed affect after four tasks (0–100)	1.40	11.61	16.00	17.89	15.33	9.77	17.18	14.00	15.32
9	Perceived task autonomy (1–5)	33.40***	3.94_a_	0.72	2.18_b_	0.87	3.68_a_	0.65	2.58_b_	0.87

The possible range of the variables is indicated between brackets. The *n* varies between 27 and 28 in the high boredom high autonomy condition, *n* = 28 in the high boredom low autonomy condition, *n* = 31 in the low boredom high autonomy condition, and *n* varies between 32 and 33 in the low boredom low autonomy condition. For variables with a significant *F-*test means which do not share the same subscript are significantly different at *p* < .05

***p < .001

To further check whether experienced state boredom and perceived task autonomy were driving the effects (rather than some other mechanism), we conducted a moderated regression analysis with frustration as dependent variable, the perceptual measures of state boredom and perceived autonomy (i.e., the manipulation checks) as predictors, and controlling for experiment duration, baseline levels of frustration, and Time 3 depressed affect. Predictor variables were mean-centered before calculating the interaction terms (cf. Aiken and West 1991). As shown in Table 5, state boredom was significantly positively related to frustration and perceived autonomy was not. Furthermore, the perceived task autonomy × state boredom interaction was significant. We further analyzed the form of the interaction using simple slopes analyses (cf. the procedures outlined by Aiken and West 1991). Supporting Hypothesis 1, state boredom related more positively to frustration at lower levels of perceived task autonomy (*M* − 1*SD*), *B* = 10.59, *SE* = 1.92, than at higher levels (*M* + 1*SD*), *B* = 1.25, *SE* = 1.84, *t*(114) = − 3.80, *p* < .001, with the low slope being significantly different from zero, *t*(114) = 5.50, *p* < .001, but the high slope not, *t*(114) = 0.68, *p* = .50.

**Table 5:** Study 2 moderated regression analyses of frustration and depressed affect after the series of four tasks on perceived task autonomy, self-reported state boredom, and their interaction

Predictor	Time 3 frustration				Time 3 depressed affect			
	Step 1		Step 2		Step 1		Step 2	
	β	*B* [95% CI]	β	*B* [95% CI]	β	*B* [95% CI]	β	*B* [95% CI]
Experiment duration	0.02	0.05 [− 0.21, 0.31]	0.01	0.01 [− 0.23, 0.26]	0.06	0.08 [− 0.05, 0.21]	0.06	0.08 [− 0.05, 0.21]
Frustration after practice	0.49***	0.63 [0.43, 0.82]	0.53***	0.67 [0.49, 0.86]				
Depressed affect after practice					0.82***	0.75 [0.67, 0.84]	0.82***	0.75 [0.67, 0.84]
Time 3 frustration					0.18***	0.13 [0.06, 0.21]	0.18***	0.13 [0.06, 0.21]
Time 3 depressed affect	0.16*	0.22 [0.01, 0.42]	0.14	0.19 [− 0.01, 0.38]				
Perceived task autonomy	− 0.09	− 1.84 [− 4.47, 0.78]	− 0.04	− 0.79 [− 3.33, 1.75]	0.01	0.13 [− 1.18, 1.43]	0.01	0.12 [− 1.21, 1.46]
State boredom	0.27***	5.69 [2.69, 8.68]	0.28***	5.92 [3.09, 8.75]	− 0.05	− 0.76 [− 2.32, 0.80]	− 0.05	− 0.76 [− 2.35, 0.82]
Perceived task autonomy × state boredom			− 0.22***	− 4.40 [− 6.70, − 2.11]			0.00	0.01 [− 1.21, 1.23]
Δ*R*^2^			0.05***				0.00	
Multiple *R*	0.77***		0.80***		0.90***		0.90***	
Adjusted *R*^2^	0.57***		0.62***		0.81***		0.81***	

*N* = 120. The possible range for scores on frustration and depressed affect was 0–100

*p < .05; ***p < .001

Lastly, we conducted path analysis in Mplus 7.11 using bootstrapping with 10,000 samples to test whether the effect of the interaction between manipulated boredom and autonomy on Time 3 frustration is explained by the interaction between experienced state boredom and perceived task autonomy. We tested a path model with direct paths to Time 3 frustration for the control variables (i.e., experiment duration, baseline frustration, Time 3 depressed affect), the manipulated variables (i.e., condition boredom, condition autonomy, and their interaction), and the perceptual measures (i.e., state boredom, perceived autonomy, and their interaction). Furthermore, we included indirect paths of the manipulated variables through their corresponding perceptual measure to Time 3 frustration. The results demonstrate that the direct effect of the condition boredom × condition autonomy interaction was not significant, *B* = − 0.35, *SE* = 1.33, *p* = .79, whereas the direct effect of the experienced state boredom × perceived task autonomy interaction was significant, *B* = − 4.25, *SE* = 1.43, *p* < .01. In addition, the indirect effect of the condition boredom × condition autonomy interaction on Time 3 frustration through the experienced state boredom × perceived task autonomy interaction was significant, *estimate* = − 1.50, 95% CI [− 2.95, − 0.05]. These findings suggest that the effects of the interaction of manipulated boredom and autonomy on Time 3 frustration are explained by the interaction of experienced state boredom and perceived task autonomy.

Hypothesis 2 was tested with a two-way ANCOVA with conditions boredom and autonomy as factors, experiment duration, baseline depressed affect (i.e., at Time 2 after the practice task) and Time 3 frustration as covariates, and depressed affect after the main task series (Time 3) as dependent variable. Results demonstrated significant main effects of depressed affect after the practice task, *F*(1, 115) = 322.38, *p* < .001, partial η^2^ = 0.74, and Time 3 frustration, *F*(1, 115) = 11.77, *p* < .001, partial η^2^ = 0.09. The main effects of experiment duration, *F*(1, 115) = 0.36, *p* = .55, partial η^2^ = 0.00, condition boredom, *F*(1, 115) = 0.01, *p* = .92, partial η^2^ = 0.00, and condition autonomy, *F*(1, 115) = 0.97, *p* = .33, partial η^2^ = 0.01, were not significant. Also, the interaction between boredom and autonomy was not significant, *F*(1, 115) = 0.10, *p* = .76, partial η^2^ = 0.00 (Hypothesis 2 not supported). Similarly, moderated regression analysis using the perceptual measures (see Table 5), indicated main effect relationships of depressed affect after the practice task and Time 3 frustration only.

## General discussion

Reactions to experiencing boredom include constructs indicative of both high and low arousal/activation levels. Previously mentioned explanations for these seemingly contradicting findings concern different stages in the boredom experience, different types of boredom, or different characteristics of the task that induces boredom (Eastwood et al. 2012; Goetz et al. 2014). In the present study we proposed another explanation, that is, conditions of the situation in which people complete a task. Under conditions of low task autonomy the state of boredom was proposed to relate to high arousal/activation levels (i.e., frustration), whereas under conditions of high task autonomy it would relate to low arousal/activation levels (i.e., depressed affect).

### Main findings

The findings of our correlational study and experimental study converge in demonstrating that the extent to which state boredom is associated with feelings of frustration depends on the level of perceived and provided autonomy during the task that people are engaging in. When having/perceiving little autonomy, boredom more likely relates to frustration than when having/perceiving more autonomy. Study 1 showed this interaction in a naturalistic setting during which people worked on a relatively boring task. Study 2 replicated this finding in a controlled setting during which boredom and autonomy were experimentally manipulated. Autonomy may thus serve as a buffer against negative high-arousal reactions to boredom, whereas having little autonomy triggers negative high-arousal reactions to boredom. These findings are consistent with theories on stress (e.g., Demerouti et al. 2001; Karasek 1979), which typically view autonomy as a resource that may buffer the negative effects of stressors and task demands.

These buffering effects of task autonomy in the context of boredom extend previous research, which demonstrated that perceiving autonomy may reduce the likelihood of experiencing state boredom (e.g., Reijseger et al. 2013; Van Hooff and Van Hooft 2017). Consistent with this research, our findings on perceived task autonomy in both studies demonstrate negative correlations with experiencing state boredom (see Tables 1, 3). Extending this research, our findings show that task autonomy not only reduces the likelihood of experiencing boredom, but also reduces subsequent negative activating affect such as feelings of frustration when boredom does occur.

Support for our expectation that autonomy also affects the relationship between boredom and depressed affect was mixed. Consistent with our expectation, Study 1 findings indicate that when perceiving high levels of autonomy, boredom associates with higher levels of depressed affect than when perceiving little autonomy. However, Study 2 findings did not support the autonomy × boredom interaction in predicting depressed affect. A potential explanation for the lack of findings regarding the moderating role of autonomy in Study 2 may relate to the context. In Study 1 the boredom inducing task consisted of a test session which was part of the psychology curriculum of students, and as such may have represented a task that is closer to the participants’ self (as psychology students), and thus more likely triggers depressed affect when this was perceived as boring. In contrast, the Study 2 task may have been interpreted more instrumentally as ‘just an experiment’ to obtain study credits or cash. Another explanation may relate to differences in task duration. Possibly for depressed affect to occur, people need to engage in boring tasks for a longer period (such as in Study 1), and possibly even more so in situations with no set and known end time. Future research should therefore test the depressed affect hypothesis in different settings (e.g., among employees in a boring job, unemployed individuals), and assess boredom and depressed affect multiple times (e.g., in a diary design) or over a longer period of time.

More generally, research is needed to further explore boredom and arousal levels. In the present study we conceptualized boredom as a transient unpleasant affective state that is related to the ongoing (lack of) activity, and proposed and found that boredom may associate with both high-arousal or low-arousal affective reactions. Rather than focusing on high versus low arousal *reactions* to boredom, other scholars have included an arousal component *within* the boredom construct space. Specifically, some authors defined boredom as a state of relatively low arousal and activation (e.g., Fisher 1993; Fisher, in press; Mikulas and Vodanovich 1993; Vogel-Walcutt et al. 2012). Others, however, referred to boredom as a restless and irritable feeling indicating high arousal and activation (e.g., Barbelet 1999; London et al. 1972; Van Tilburg and Igou 2012), or as an emotion that can vary in level of arousal (Eastwood et al. 2012; Goetz and Frenzel 2006; Goetz et al. 2014). For example, Goetz et al. (2014) distinguished between various types of boredom along the valence × arousal space (e.g., indifferent boredom, apathic boredom, reactant boredom) and tested this model in an experience sampling design among students. Specifically, participants were asked about their current activity, and when they experienced boredom they were asked how it feels to be bored and rated this on an arousal scale from 1 (*calm*) to 5 (*fidgety*). Based on this research design, it is hard to determine whether the arousal actually is a component of boredom per se, or a reaction to boredom. Future research is therefore needed to further examine whether there are actually different types of boredom or whether these reflect different reactions to boredom depending on the task and situation.

Furthermore, other situational characteristics apart from autonomy may relate to high- versus low-arousal reactions to boredom. For example, task characteristics that are important for motivation (e.g., task identity; Hackman and Oldham 1980) or time-related factors such as time pressure or the time left on the task may impact the relationship of boredom with arousal level. When people experience boredom and they have high (rather than low) task identity or the (task) situation is infinite (rather than finite), it might more likely trigger depressed affect because the situation may feel important for the self or may feel hopeless. Future research should also include the reasons for being bored, as this may differ depending on task characteristics (i.e., having nothing to do, doing a repetitive task, doing a passive task). These task characteristics potentially might determine the subsequent arousal levels associated with boredom. Also the context surrounding the boredom-inducing task may impact the relationship of boredom with arousal level. For example, boredom might more likely trigger frustration when people have an important alternative task/goal that is obstructed by the boredom-inducing task, whereas it might more likely trigger depressed affect when people have no other important conflicting tasks/goals. In addition, future research may explore whether individual differences such as locus of control, trait procrastination, or conscientiousness may affect the reactions to boredom. For example, people with an internal rather than an external locus of control might be more likely to alter the situation when experiencing boredom, thus leading to less negative reactions to boredom. Those high on trait procrastination may linger in the boring task, triggering low arousal, whereas those high on conscientiousness may want to finish the boring task more quickly, triggering high arousal.

### Limitations and conclusion

Some limitations should be taken into account when interpreting our findings. A first limitation may be that our manipulation checks in Study 2 indicated the presence of interactive effects between manipulated boredom and autonomy on the Time 3 self-report state boredom and perceived autonomy measures. However, concerns that may be raised because of these interactions are alleviated by the following findings. That is, simple effects analyses to interpret the interactions showed that the manipulation of boredom was effective in both autonomy conditions, and that the manipulation of autonomy was effective in both boredom conditions. Moreover, our regression analyses using the manipulation check measures demonstrated further support for Hypothesis 1, and the subsequent path analyses indicated that the effect of the boredom × autonomy interaction on frustration may be explained by the interaction between the manipulation check measures of state boredom and perceived autonomy.

A second study limitation relates to the samples and setting. Both studies involved student samples, with relatively more females, in a setting where the boring tasks are known to last for a relatively short time (i.e., 2.25 h in Study 1 and about 45 min in Study 2). Furthermore, in Study 1 participants were required to complete the measures as part of their study program. Participation in Study 2 was voluntary, and participants received an incentive (i.e., study credit or €7 cash). These task settings may have influenced participants’ situational motivation. Future research should therefore test the generalizability of our findings in different samples and contexts, and using different tasks.

A third limitation relates to the measures. Some of our measures had relatively low alphas (i.e., task autonomy) and all measures relied on relatively short self-report scales. To reduce issues associated with self-report measures such as socially desirable responding, we emphasized the participants’ anonymity. Furthermore, it should be noted that in Study 2 the independent variables (i.e., state boredom and task autonomy) were manipulated rather than measured. Nevertheless, future research may examine the role of autonomy in the associations of boredom with frustration and depressed affect using structural equation modelling with latent factors, using other more elaborate measures, or using non self-report measures (e.g., physiological measures) for assessing arousal levels. In addition, future research should examine whether feelings of frustration explain the effects of boredom on more distal outcomes such as aggression and counterproductive behavior.

Despite the limitations, our findings may have important practical implications (e.g., for educators, managers). Because boredom may trigger frustration or potentially depressed feelings, depending on task autonomy, in practice prevention of boredom is essential. This can be achieved, for example, by designing the work or educational context in such a way that it contains sufficient skill variety (i.e., allow students/employees to use different skills), or task identity (i.e., allowing students/employees to perform a whole piece of work from beginning to end; Fisher 1993; Loukidou et al. 2009; Van Hooff and Van Hooft 2017). Also providing an environment in which students or employees have the opportunity to fulfill their basic psychological needs to feel competent and related to other people (Deci and Ryan 2000) may be beneficial in this respect (Van Hooff and Van Hooft 2017). This can be achieved, for example, by providing constructive feedback or supporting social interactions between students/employees. In the work context boredom may be reduced by encouraging employees to engage in job crafting (Harju et al. 2016; Van; Hooff and Van Hooff 2014), which enables them to align their work tasks with their personal abilities and needs. However, when tasks that are relatively boring (e.g., monotonous, too easy, repetitive) need to be done, careful attention is warranted. In order to reduce frustration caused by such tasks, substantial autonomy should be provided. Autonomy can be supported by providing meaningful rationales for engaging in a task, minimizing the use of contingent rewards and punishments, providing opportunities for participation and choice where possible, and acknowledging negative feelings associated with boring tasks (cf. Deci et al. 1994; Oliver et al. 2008). However, close monitoring is needed that the combination of boredom and increased levels of autonomy does not trigger depressed affect. In case of heightened levels of depressed affect in response to boredom-inducing tasks, these tasks should be alternated with tasks that fulfill people’s basic psychological needs for competence and relatedness (Van Hooff and Van Hooft 2016).
